# Development and external validation of machine-learning based models to predict diabetic foot ulcer in diabetes population

**DOI:** 10.3389/fendo.2025.1692917

**Published:** 2025-12-15

**Authors:** Yanji Zhang, Yingying Tian, Yang Jian, Zairong Wei, Silang Cai, Gaofengi Zhang, Chengliang Deng

**Affiliations:** 1 Department of Burns and Plastic Surgery, Affiliated Hospital of Zunyi Medical University, Zunyi, Guizhou, China; 2 Department of Radiology, The Second Affiliated Hospital of Zunyi Medical University, Zunyi, Guizhou, China; 3 Department of Medical Education, Kweichow Moutai Hospital, Zunyi, Guizhou, China

**Keywords:** machine learning, diabetic foot ulcer, NHANES, external validation, diagnosis

## Abstract

**Background:**

Diabetic foot ulcer (DFU) is a common and serious complication in patients with diabetes, which affects the quality of life greatly as well as brings high risk for mortality. Identification of high-risk individuals, as early as possible is important for efficient intervention and prevention. This study systematically evaluates and summarizes the diagnostic accuracy of machine learning approaches for predicting DFU risk in diabetic patients.

**Methods:**

This study adhered to the TRIPOD+AI (Transparent Reporting of a Multivariable Prediction Model for Individual Prognosis or Diagnosis, Extended for Artificial Intelligence) guidelines. Using data from the National Health and Nutrition Examination Survey (NHANES) 1999–2004 to determine diagnosis of DFU related clinical characteristics, laboratory indicators and lifestyle-related variables. The diagnostic performance of its models trained using Logistic Regression (LR), K-Nearest Neighbors (KNN), Random Forest (RF), Extreme Gradient Boosting (XGBoost) and Support Vector Machine (SVM) classifiers were compared. An independent testing dataset collected from the Second Affiliated Hospital of Zunyi Medical University was used to conduct external validation.

**Results:**

This study included 1, 857 participants from NHANES and 807 individuals recruited at the testing dataset. Key predictors identified in NHANES were numbness in extremities, direct HDL cholesterol, lymphocyte, white blood cell, segmented neutrophils, and BMI. Among them, the RF was identified as having the highest area under receiver operating characteristic curve (AUC) for NHANES at 0.81. The RF model also had the highest discriminative performance in external validation (as measured by an AUC of 0.79). Other models also provided good results in external validation: XGBoost had an AUC of 0.76, SVM reached 0.72, KNN reached 0.70, and LR received a score of 0.69.

**Conclusion:**

The ability of machine learning models to predict DFU risk was good in a combined population cohort when measured using common metrics but varied across distinct regions. These results support future clinical evaluation of these models and underscore the need to select algorithms *a priori* based on the target patient population.

## 1 Introduction

Diabetes mellitus is a chronic metabolic disorder, which has remained as one of the largest global public health problems steadily increasing prevalence. An estimated 463 million adults live with diabetes globally and, without effective prevention strategies in place the number is likely to rise to 693 million by the year of 2045 (1). The data show China at the top of global rankings, with some 116.4 million sufferers overall. About 40% population of diabetics is unaware of their disease; moreover, companion disorders are chronic in the same way source (2). Complications such as cardiovascular disease, nephropathy, retinopathy, and diabetic foot ulcer (DFU) largely contribute to the risk of disability, death, and healthcare burden (3).

DFU is one of the serious chronic diabetic complications, usually involuing skin breakdown, infection, and even deep tissue destruction in the foot, mainly caused by neuropathy and microvascular disease (4). Worldwide, the risk of lower limb amputation in patients with DFU throughout their lifetime is as high as 20%, and 5-year mortakity rate is between 50% and 70% (5). In China, DFU prevalence is still on the increase, and most cases are only diagnosed at advanced stages, resulting in increased recurrence and amputation rates (6). Delayed recognition and intervention greatly elevate the risks of infection, gangrene, amputation, and even death, seriously affecting patients’ quality of life and causing a heavy healthcare burden (7, 8). Thus, the early detection of individuals at high risk and timely, personalized intervention are of great importance for ulcer prevention and outcome inprovement in patients.

The development of foot ulcer in patients with diabetes is a complex phenomenon involving both intrinsic and extrinsic factors. Existing literature has shown that the prevalence of DFU is closely associated with a constellation of clinical characteristice, biochemical markers, and lifestyle factors (9). Evidence has demonstrated that patients with diabetic retinopathy have a 1.74 time higher risk of developing DFU, whereas peripheral vascular disease has been identified as an independent risk factor for DFU in particular among Asian populations (10). A large-scale observational study in Europe also provided further evidence that improvement in glycemic and lipid control in patients with type 1 diabetic (T1D) and the elimination of smoking and alcohol consumption in patients with type 2 diabetes (T2D) were effective strategies to reduce the risk of DFU (11). In addition, numerous studies have reported that increased concentrations of HbA1c and C-reactive protein (CRP) were positively correlated with the severity of DFU-as reflected by increased Wagner grades-and were significantly associated with an elevated risk of DFU (12; 13).

Considering the multifactorial nature of DFU and the growing availability of clinical and biochemical datasets, machine learning (ML) methods have emerged as promising tools for integrating complex risk factors and strengthening early risk diagnosis (14). Compared with traditional statistical methods, ML algorithms have better ability in handling high-dimensional data, detecting nonlinear relationships, and optimizing diagnostic efficiency (15). Previous research has initially suggested the prospects of ML models in predicting the risk of DFU. However, limitations like reliance on single data types (e.g., clinical characteristics or images), inability to incorporate multidimensional variables, and absence of external validation have hindered the generalizability of such models to diverse populations (16; 17).

The purpose of this research is to determine DFU risk factors based on the 1999–2004 NHANES data and to construct ML models to predict DFU risk. Both model generalizability and clinical usefulness were tested with an external dataset. Through the incorporation of multidimensional variables and the comparing of algorithm performance, it lays the groundwork for the early detection and indivdualized risk determination of DFU.

## 2 Materials and methods

### 2.1 Study design and population

This study was conducted and reported in accordance with the TRIPOD+AI (Transparent Reporting of a Multivariable Prediction Model for Individual Prognosis or Diagnosis, Extended for Artificial Intelligence) guidelines to ensure transparency and clarity in the development and validation of the prediction model (18). This research used information from two sources: the National Health and Nutrition Examination Survey (NHANES) and a separate clinical dataset from the Second Affiliated Hospital of Zunyi Medical University in China.

**Figure 1:**
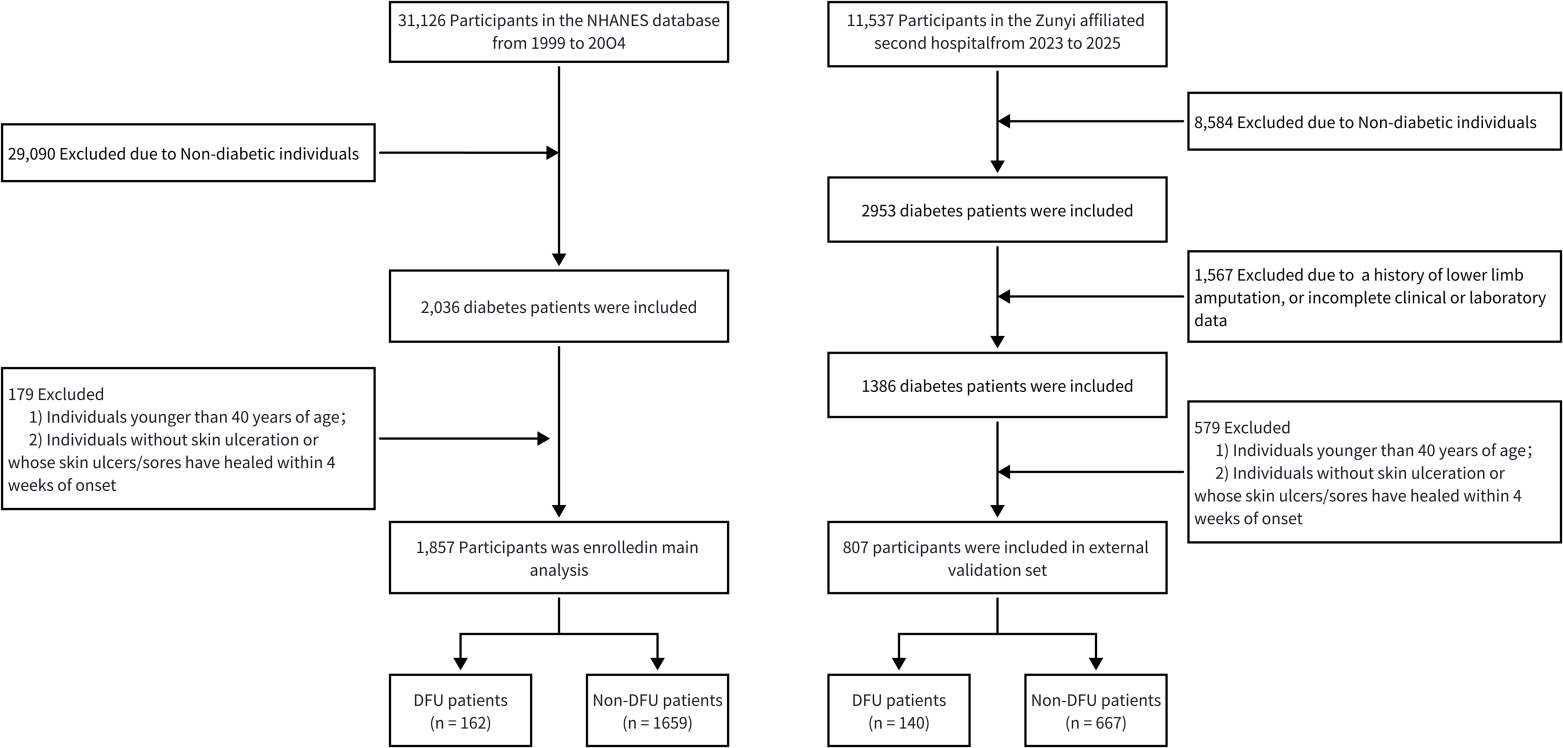
Flow chart of subject inclusion and exclusion.

NHANES is a nationally representative survey conducted biennially by the National Center for Health Statistics (NCHS) to assess the health and nutritional status of the U.S. population through household interviews and mobile examination centers. The program was approved by the NCHS Ethics Review Board, and all participants provided written informed consent. For this study, NHANES data from 1999 to 2004 were used, yielding an initial sample of 31, 126 participants. Participants without diabetes, those under 40 years of age, and individuals without skin ulceration or whose ulcers/sores had healed within 4 weeks of onset were excluded from the study. (**Figure 1**). More information about NHANES is available at: https://wwwn.cdc.gov/nchs/nhanes.

Clinical data of diabetic patients who were admitted to the Second Affiliated Hospital of Zunyi Medical University between June 2023 and May 2025 were retrospectively collected for the creation of the external validation cohort. To ensure consistency, participants were selected based on the same inclusion criteria set for the NHANES dataset. Additionally, patients who had foot ulcers due to non-diabetic causes (like trauma, autoimmune diseases, or malignancies), a reported histort of lower limb amputation, or incomplete clinical or laboratory data were excluded from the study. The study was approved by the relevant institutional ethics committee (No. KYLL-2025-128), and written informed consent was obtained from all participants.

### 2.2 Definition of diabetes and diabetic foot ulcers

Participants were classified as having diabetes mellitus if they met at least one of the following criteria: 1) glycohemoglobin (HbA1c) of ≥ 6.5%; 2) random blood glucose of ≥ 11.1 mmol/L; 3) fasting blood glucose of ≥ 7.0 mmol/L; 4) current use of anti-glycemic drugs; or 5) a prior diagnosis of diabetes made by a healthcare provider. DFU was harmonized across cohorts using the “unhealed ≥4 weeks” construct. In NHANES, DFU was self-reported; in the external validation cohort, DFU was clinician-verified according to the Chinese Guidelines for the Diagnosis and Treatment of Diabetic Foot, using the same ≥ 4-week observation window (19–21).

### 2.3 Data collection

Data were collected from both cohort in four different domains: demographics including age, gender, and body mass index (BMI); medical history such as hypertension, hypercholesterolemia, coronary heart disease (CHD), stroke, retinopathy, numbness in hands or feet, leg pain while walking, and kidney weakness; lifestyle behaviors including smoking and alcohol consumption; and laboratory parameters such as HbA1c, fasting blood glucose, random blood glucose, total cholesterol, direct high-density lipoprotein cholesterol (HDL-C), urine creatinine, urine albumin, serum creatinine, albumin, total calcium, phosphorus, potassium, CRP, white blood cell count (WBC), segmented neutrophils, lymphocytes, and hemoglobin. Detailed definitions and descriptions of all study variables were provided in **Supplementary Table S1**.

### 2.4 Statistical analysis

The analyses were run separately using R version 4.2.2 and Python version 3.12. Predictors were processed with one-hot encoding for categorical variables and z-score standardization for continuous variables; ‘don’t know/refused’ were treated as missing after unit harmonization and LOD code handling. The continuous variables were reported as means ± standard deviations (SD), and categorical variables were reported as proportions. T-tests for continuous variables and χ2 tests for categorical variables were used to etermine baseline differences between DFU participants and non-DFU participants. Variables significant in univariate analyses were entered into a multivariable logistic regression to identify independent risk factors, from which coefficients, odds ratios (ORs), 95% confidence intervals (CIs), and p-values were estimated. The statistical significance level was set at P < 0.05.

To develop and evaluate predictive models, five machine learning algorithms were used, such as logistic regression (LR), random forest (RF), extreme gradient boosting (XGBoost), k-nearest neighbors (KNN), and support vector machine (SVM). The NHANES dataset was subjected to random splitting into a training set of 70% and an internal validation set of 30%. Missing values were imputed by mode for categorical variables and by median for continuous variables (**Supplementary Table S2**). Hyperparameter optimization was carried out on the training dataset using stratified 5-fold cross-validation: grid search for LR, RF, SVM, KNN, and XGBoost, and randomized search followed by a reduced-grid search for XGBoost. To counter class imbalance, class weights (and scale_pos_weight for XGBoost) were used, achieving better performance without risking severe overfitting (**Supplementary Table S3**). The performance of the models was evaluated on the internal validation set and external validation set using several metrics that included the area under the receiver operating characteristic curve (AUC), accuracy, sensitivity, specificity, precision, and F1 score. The optimal threshold was determined by maximizing Youden’s J statistic to achieve the best balance between sensitivity and specificity. In addition, decision curve analysis and calibration curve methodology were used to evaluate the clinical usefulness and the agreement between the predicted and actual outcomes of the models, respectively.

## 3 Results

### 3.1 Participant characteristics

**Table 1** shows the baseline characteristics of all participants included. Overall, 2, 664 patients were enrolled, of whom 1, 857 were from the NHANES cohort (162 with DFU and 1, 695 without DFU) and 807 were from the external validation cohort (140 with DFU and 667 without DFU). Within the NHANES cohort, no significant between-group differences were noted for age (65.37 ± 11.95 vs. 65.20 ± 11.94 years) or gender distribution (male: 56.17% vs. 50.86%, P = 0.23) between the DFU and non-DFU groups. The DFU group, however, had significantly higher prevalence rates of retinopathy, numbness in the extremities, pain in the legs when walking, impaired renal function, and abnormal levels of a number of inflammation- and kidney-associated biomarkers (e.g., urine albumin, segmented neutrophils, creatinine). In the external validation cohort, DFU patients tended to be slightly older than non-DFU patients (62.03 ± 11.35 vs. 60.14 ± 8.97 years), and the percentage of males was similarly slightly higher (58.57% vs. 49.78%), although neither of these differences was statistically significant. Relative to the non-DFU group, DFU patients had worse metabolic and vascular profiles, with higher prevalence rates of retinopathy, neuropathy, stroke, and hypertension, as well as significant abnormalities in laboratory measures such as glycohemoglobin, CRP, creatinine, and hemoglobin.

**Table 1:** Characteristics of study participants.

Variable	NHANES cohort			External validation cohort		
	DFU (n = 162)	Non-DFU (n = 1695)	*P* value	DFU (n = 140)	Non-DFU (n = 667)	*P* value
Age	65.37 ± 11.95	65.20 ± 11.94	0.86	62.03 ± 11.35	60.14 ± 8.97	0.07
Gender			0.23			0.07
Male	91 (56.17%)	862 (50.86%)		82 (58.57%)	332 (49.78%)	
Female	71 (43.83%)	833 (49.14%)		58 (41.43%)	335 (50.22%)	
BMI	32.12 ± 7.74	30.95 ± 6.54	0.10	25.71 ± 3.87	24.85 ± 3.08	**0.02**
Retinopathy	67 (41.35%)	323 (19.05%)	**<0.01**	99 (70.71%)	300 (44.98%)	**<0.01**
Numbness in extremities	104 (64.20%)	528 (31.15%)	**<0.01**	116 (82.86%)	270 (40.48%)	**<0.01**
Leg pain while walking	90 (55.55%)	620 (36.58%)	**<0.01**	41 (29.29%)	51 (7.65%)	**<0.01**
Stroke	19 (11.73%)	161 (9.50%)	0.57	26 (18.57%)	53 (7.95%)	**<0.01**
CHD	26 (16.05%)	194 (11.45%)	0.15	46 (32.86%)	214 (32.08%)	0.94
Hypertension	108 (66.66%)	1078 (63.60%)	0.30	71 (50.71%)	269 (40.33%)	**0.03**
High Cholesterol	83 (51.23%)	774 (45.66%)	0.68	43 (30.71%)	317 (47.53%)	**<0.01**
Renal dysfunction	35 (21.60%)	129 (7.61%)	**<0.01**	37 (26.43%)	31 (4.65%)	**<0.01**
Alcohol consumption	80 (49.38%)	867 (51.15%)	0.15	38 (27.14%)	237 (35.53%)	0.07
Smoking	88 (54.32%)	895 (52.80%)	0.82	68 (48.57%)	270 (40.48%)	0.10
Glycohemoglobin	7.63 ± 2.15	7.43 ± 1.78	0.28	13.54 ± 5.17	8.06 ± 0.84	**<0.01**
Fasting blood glucose, mmol/L	9.83 ± 4.56	9.07 ± 3.67	0.21	14.39 ± 6.41	13.95 ± 4.44	0.44
Random blood glucose, mmol/L	9.22 ± 5.16	8.31 ± 3.99	0.11	14.08 ± 5.08	13.08 ± 4.47	**0.03**
Total Cholesterol, mmol/L	5.12 ± 1.20	5.31 ± 1.26	0.09	4.16 ± 1.29	4.79 ± 0.98	**<0.01**
Direct HDL-Cholesterol, mmol/L	1.24 ± 0.45	1.23 ± 0.36	0.72	1.07 ± 0.48	1.16 ± 0.27	0.051
Urine creatinine, mg/dL	109.41 ± 73.52	116.20 ± 74.75	0.31	79.85 ± 48.52	86.38 ± 41.13	0.139
Urine albumin, ug/mL	465.15 ± 1297.71	209.93 ± 987.54	**0.03**	319.69 ± 710.69	56.96 ± 200.88	**<0.01**
WBC, 10³/µL	7.81 ± 2.66	7.48 ± 2.15	0.17	7.92 ± 3.33	6.65 ± 1.68	**<0.01**
Segmented neutrophils, 10^9^/L	4.88 ± 2.11	4.47 ± 1.64	**0.03**	5.53 ± 3.30	4.15 ± 1.46	**<0.01**
Lymphocyte, 10^9^/L	2.04 ± 0.90	2.18 ± 0.90	0.09	1.52 ± 0.59	1.74 ± 0.52	**<0.01**
Hemoglobin, g/dL	13.67 ± 1.62	14.13 ± 1.60	**<0.01**	11.01 ± 2.22	13.75 ± 1.83	**<0.01**
Potassium, mmol/L	4.26 ± 0.44	4.10 ± 0.41	**<0.01**	4.17 ± 0.51	4.02 ± 0.32	**0.01**
Total calcium, mmol/L	2.36 ± 0.13	2.37 ± 0.11	0.39	2.22 ± 0.18	2.33 ± 0.13	0.25
Phosphorus, mmol/L	1.22 ± 0.20	1.17 ± 0.20	**0.01**	1.19 ± 0.35	1.16 ± 0.24	0.39
Albumin, g/dL	4.04 ± 0.36	4.18 ± 0.35	**<0.01**	4.74 ± 1.65	4.05 ± 0.38	**<0.01**
Creatinine, umol/L	120.93 ± 133.38	87.08 ± 63.18	**0.01**	138.46 ± 152.19	76.55 ± 42.62	**<0.01**
CRP, mg/dL	0.84 ± 1.21	0.72 ± 1.51	0.26	2.65 ± 3.37	0.71 ± 1.43	**<0.01**

DFU, diabetic foot ulcers; BMI, body mass index; CHD, coronary heart disease; HDL, high-density lipoprotein; WBC, white blood cell; CRP, C-reactive protein.

Footnote: Bold values indicate statistically significant differences.

### 3.2 Logistic regression analysis

In order to detect independent predictors of DFU, we conducted univariate and multivariate logistic regression analysis (**Table 2**). Six variables were independently related: numbness in the extremities (Coef = 1.34, OR = 3, 82, 95% CI: 2.68–5.49, P < 0.01), direct HDL cholesterol (Coef =1.54, OR = 4.65, 95% CI: 1.66-13.02, P < 0.01), lymphocyte (Coef = -2.76, OR = 0.06, 95% CI: 0.01-0.14, P = 0.01), WBC (Coef = 2.03, OR = 7.63, 95% CI: 1.54-37.77, P = 0.01), segmented neutrophils (Coef = -0.91, OR = 0.40, 95% CI: 0.30-0.54, P = 0.02), and BMI (Coef = 0.07, OR = 1.07, 95% CI: 1.01-1.14, P = 0.04).

**Table 2:** Multivariate logistic regression analysis of risk factors based on NHANES cohort.

Variable	Coef	OR	95% CI		P-value
Numbness in extremities	1.34	3.82	2.68	5.49	< 0.01
Direct HDL Cholesterol	1.54	4.65	1.66	13.02	< 0.01
Lymphocyte	-2.76	0.06	0.01	0.14	0.01
White blood cell count	2.03	7.63	1.54	37.77	0.01
Segmented neutrophils	-0.91	0.40	0.30	0.54	0.02
Body mass index	0.07	1.07	1.01	1.14	0.04

Coef, Coefficient; OR, Odds Ratio; CI, Confidence Interval.

### 3.3 Development and validation of ML models

**Figure 2:**
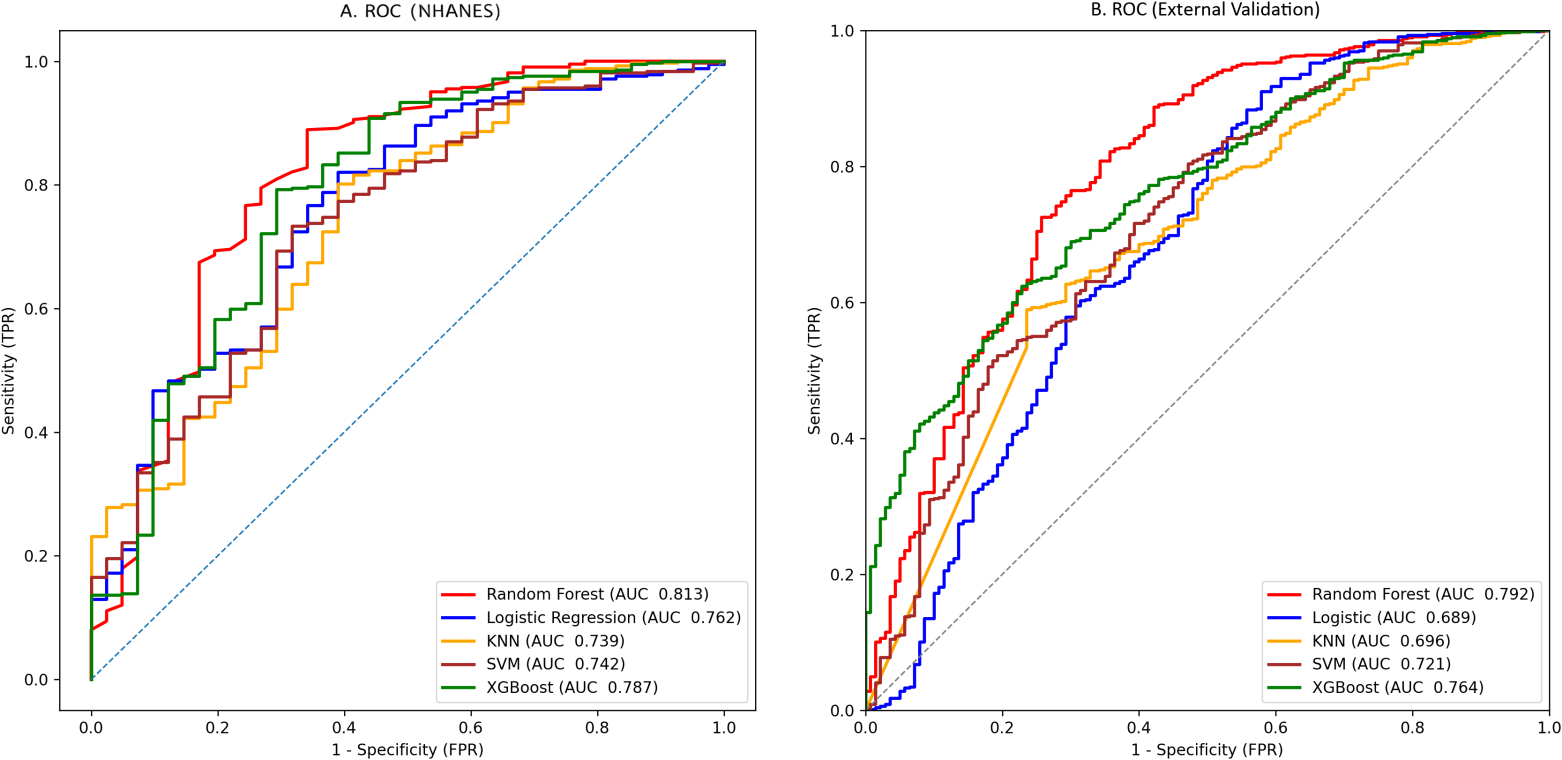
Prediction performance by AUCs for the five machine-learning models: **(a)** NHANES cohort; **(b)** external validation cohort.

**Figure 3:**
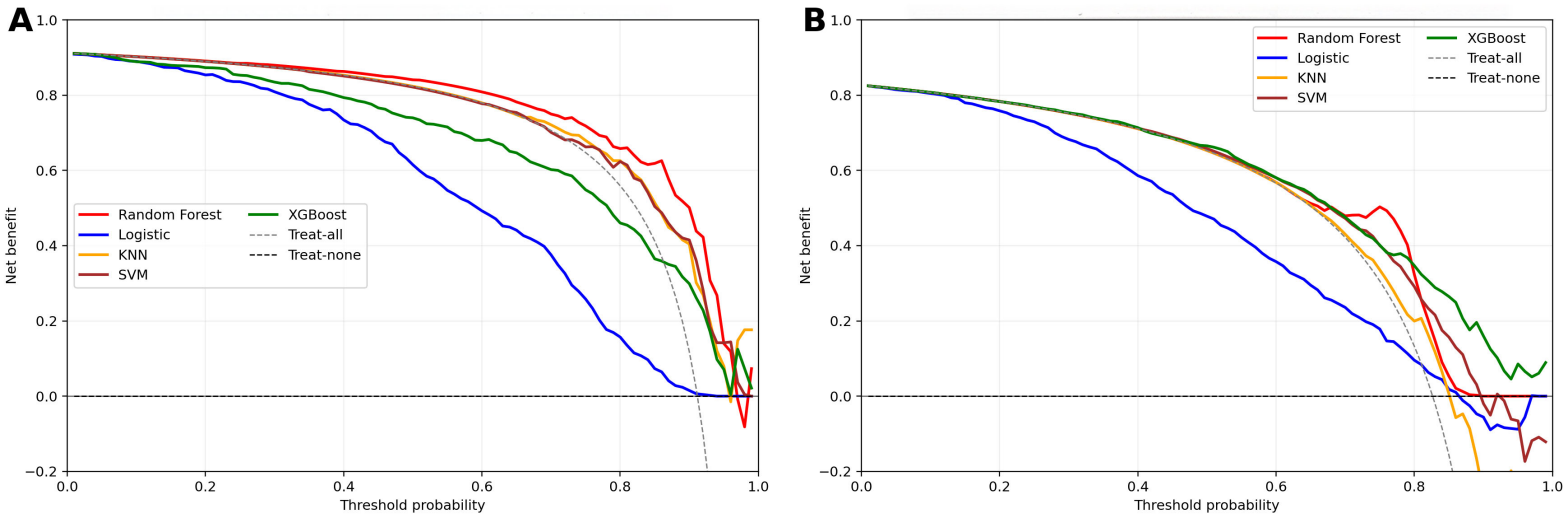
Decision curve analysis for clinical models in two cohorts. **(a)** NHANES cohort; **(b)** External validation cohorts; Net benefit is plotted against threshold probability; dashed lines denote “treat-all” and “treat-none”.

**Figure 4:**
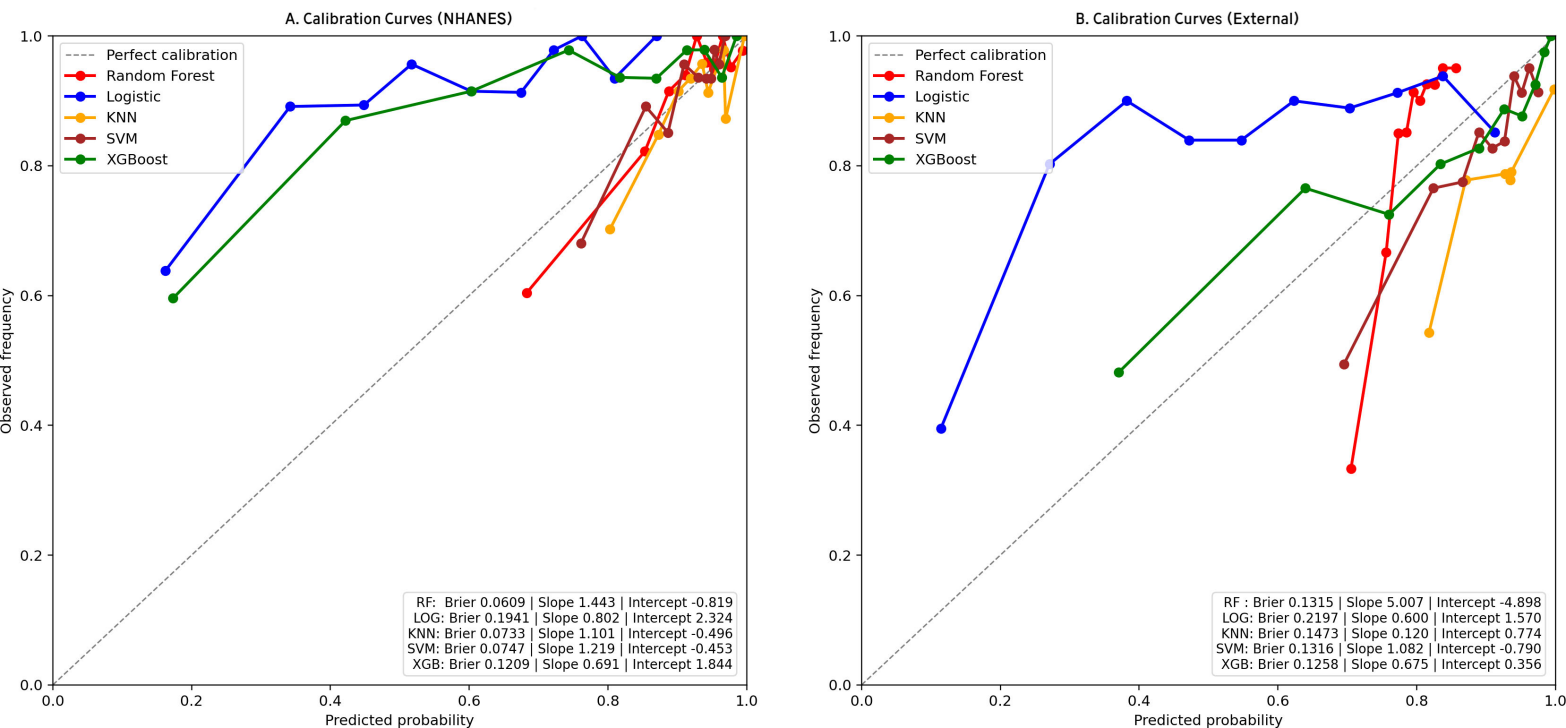
Calibration curves for clinical models in two cohorts. **(a)** NHANES cohort; **(b)** External validation cohorts. Observed outcome frequency versus predicted probability; the 45° line indicates perfect calibration.

We trained 5 machine learning models on the NHANES dataset and internally validated their predictive performance (**Table 3**, **Figure 2a**). The results indicated that, RF had the highest AUC (0.81, 95% CI: 0.76-0.87), followed by XGBoost (0.79, 95% CI: 0.73-0.85), LR (0.76, 95% CI: 0.70-0.82), KNN (0.74, 95% CI: 0.67-0.81), and SVM (0.74, 95% CI: 0.68-0.8). **Supplementary Figure S1** shows the top ten features for the RF model on the NHANES dataset, including BMI, lymphocyte count, numbness in extremities, and diabetic retinopathy, among others. The complete search spaces and the best hyperparameters for all models are summarized in **Supplementary Table S3**. The model correctly identified 27 DFU cases (true positives) and 377 non-DFU cases (true negatives). These findings suggest that the RF model exhibits strong predictive capacity in detecting DFU risk (**Supplementary Figure S2a**). Decision curve analysis resulted in positive net benefit for the RF model for 5–20% thresholds, signifying its use utility (**Figure 3a**). The calibration curve for RF model had acceptable agreement along with significant calibration slope and intercept, denoting Brier score 0.06, calibration slope 1.44, and calibration intercept -0.82. (**Figure 4a**).

**Table 3:** Performance comparison of DFU prediction models using NHANES and external validation cohorts.

Dataset	AUC (95% CI)	Accuracy	Sensitivity	Specificity	Precision	F1 Score	Best Threshold
Logistic Regression							
NHANES cohort	0.76 (0.70-0.82)	0.80 (0.76-0.84)	0.82 (0.78-0.852)	0.61 (0.46-0.74)	0.96	0.88	0.42
External Validation	0.69 (0.65-0.73)	0.83 (0.80-0.85)	0.91 (0.89-0.93)	0.42 (0.34-0.50)	0.88	090	0.27
Random Forest							
NHANES cohort	0.81 (0.76-0.87)	0.87 (0.84-0.90)	0.89 (0.86-0.92)	0.66 (0.51-0.78)	0.96	0.93	0.86
External Validation	0.79 (0.76-0.83)	0.73 (0.70-0.76)	0.73 (0.69-0.76)	0.74 (0.66-0.81)	0.93	0.82	0.79
XGBoost							
NHANES cohort	0.79 (0.73-0.85)	0.79 (0.75-0.82)	0.79 (0.75-0.83)	0.71 (0.56-0.82)	0.97	0.87	0.61
External Validation	0.76 (0.73-0.80)	0.65 (0.62-0.68)	0.62 (0.59-0.66)	0.77 (0.70-0.83)	0.93	0.75	0.89
K-Nearest Neighbors							
NHANES cohort	0.74 (0.67–0.81)	0.79 (0.75-0.82)	0.80 (0.76-0.84)	0.61 (0.46-0.74)	0.96	0.87	0.90
External Validation	0.70 (0.65–0.74)	0.62 (0.59–0.65)	0.59 (0.55–0.63)	0.76 (0.69–0.83)	0.92	0.72	0.94
Support Vector Machine							
NHANES cohort	0.74 (0.68–0.81)	0.73 (0.69-0.77)	0.74 (0.69-0.77)	0.68 (0.53-0.80)	0.96	0.83	0.90
External Validation	0.72 (0.68–0.76)	0.76 (0.73–0.78)	0.80 (0.77–0.83)	0.53 (0.45–0.61)	0.89	0.85	0.87

### 3.4 External validation of ML models

In the external validation set, RF yielded the highest AUC (0.79, 95% CI: 0.76-0.83), followed by XGBoost (0.76, 95% CI: 0.73-0.80), SVM (0.72, 95% CI: 0.68–0.76), KNN (0.70, 95% CI: 0.65-0.74), and LR (0.69, 95% CI: 0.65-0.73). Although KNN and SVM showed high sensitivity (0.844 and 0.845, respectively), RF exhibited the best overall discriminative performance in the external validation cohort according to AUC(**Table 3**, **Figure 2b**). Confusion matrix analysis indicated that the RF model accurately identified 104 DFU cases (true positives) and 484 non-DFU cases (true negatives). These findings indicate that the RF model exhibits strong specificity and overall classification performance in identifying individuals at risk for DFU (**Supplementary Figure S2b**). The RF model likewise maintained positive net benefit over 5–20% thresholds, supporting clinical utility (**Figure 3b**). The calibration curve of the RF model showed agreement with some degree of deviation from ideal calibration: Brier score 0.13, calibration slope 5.01, calibration intercept −4.90 (**Figure 4b**).

### 3.5 SHAP-based model interpretability analysis

**Figure 5:**
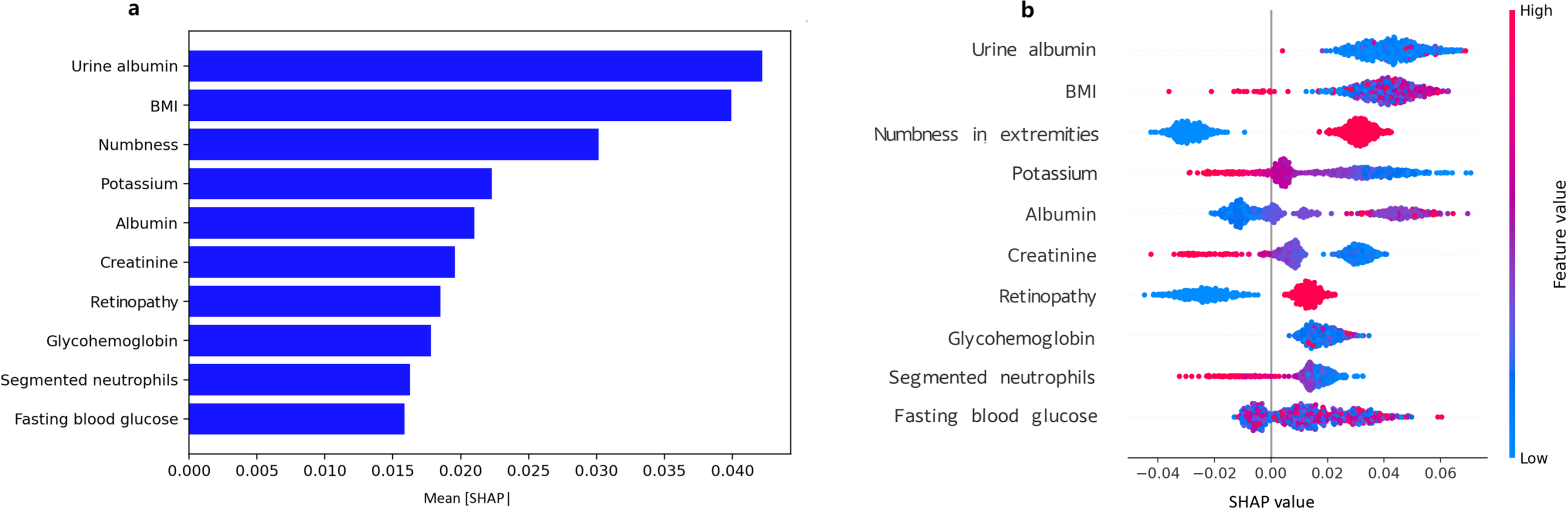
SHAP diagram for predicting DFU using the random forest model. **(a)** SHAP bar plot of feature importance, Mean absolute SHAP values rank features by global importance; **(b)** SHAP summary plot of feature importance, Each point shows a patient-level SHAP value (red = higher feature value, blue = lower).

The SHAP analysis was carried out for assessing the contribution and significance of each feature to the RF model. The top 10 features contributing to the RF model were, in descending order, urine albumin, BMI, numbness in extremities, potassium, serum albumin, creatinine, retinopathy, HbA1c, segmented neutrophils, and fasting blood glucose (**Figure 5a**). The top features tend to include positively valued SHAP for urine albumin, BMI, numbness, and glycemic parameters (HbA1c and fasting glucose) indicative of higher risk for DFU, and negatively valued SHAP for serum albumin, showing a protective association. The features for potassium, creatinine, and segmented neutrophils tend to exhibit mixed direction association (**Figure 5b**).

## 4 Discussion

Our results showed that DFU has partially independent associations with several important factors, namely the numbness of limbs, direct HDL-cholesterol, lymphocyte, white blood cell, segmented neutrophils, and BMI. The risk of DFU increases with numbness in limbs, higher levels of direct HDL-cholesterol, white blood cell, and BMI, and is strongly positively related to the white blood cell count, while it is inversely related to the lymphocyte and segmented neutrophils. The RF model has the highest performance for both internal and external validation, and the decision curve analysis clearly identified net benefit for both 5% - 20% thresholds. The calibration curve of the RF model showed acceptable overall agreement but systematic calibration bias, especially in the external validation, where the high calibration slope (5.01) suggests model overconfidence in low-risk predictions. SHAP values for analysis identified that the important factors for predicting DFU include variables like urine albumin, BMI, numbness in limbs, potassium, and albumin.

To date, several studies have attempted to build risk-prediction models for DFU. Researchers constructed a conventional logistic regression model based on five predictors, such as BMI and foot signs, which showed good performance with an AUC of 0.787 in internal validation (22). Chen et al. employed LASSO and logistic regression to screen out significant risk factors and constructed a nomogram prediction model for DFU in older patients with type 2 diabetes, with an AUC of 0.851 in internal validation (23). Another study, based on a large patient cohort with type 2 diabetes, developed and validated a diabetic foot risk score prediction model by extracting risk ratios through systematic review and meta-analysis, and adopting a weighter scoring approach to nine clinical factors, showing good discriminative ability (24). In addition to conventional model-based research, recent studies have increasingly resorted to machine learning techniques. Two studies based on data from type 2 diabetes patients in Malaysia adopted several ML methods to construct prediction models for DFU, and both showed that ML models consistently outperformed conventional methods (25; 26). Our results confirm previous work, offering additional validation across regions (NHANES, a cohort study in China, demonstrating greater generalizability for RF). Our study presents operating points for deployment (thresholds from confusion matrices) for better interpretation using SHAP, pointing towards factors for improvement (**Supplementary Table S4**).

Our multivariate logistic regression found six independent predictors of DFU: numbness in extremities, direct HDL cholesterol, lymphocyte, white blood cell, segmented neutrophils, and BMI. Numbness in the extremities, being a characteristic sign of diabetic peripheral neuropathy, is positively correlated with DFU most strongly, indicating multisystem nerve damage that compromises protective sensation and skin integrity, thus predisposing to a high risk of foot ulceration and poor healing (27). Direct HDL cholesterol can also undergo glycation and oxidation, forming “dysfunctional HDL, “ disrupting the function of reverse cholesterol efflux and endothelial and anti-inflammatory properties, leading to compromised microvascular perfusion, thereby contributing to the development and progression of DFU (28). Anemia can indicate lymphopenia, thereby disrupting the body’s ability to properly modulate the immune system, leading to inflammation, impaired cell proliferation and differentiation, and then impaired remodeling, contributing to the delayed healing process, thereby increasing the risk for DFU (29). An elevated white blood cell level can indicate chronic inflammation or occult infection, thereby increasing the risk for microvascular endothelial and cell damage, leading to inflammation, oxidative damage, and thereby increasing the risk for DFU (30). An abnormal number of segmented neutrophils can indicate a predisposition to altered cellular functions, specifically impaired chemotaxis and impaired or diminished phagocytosis, excessive production of NET, thereby predisposing the body to inflammation, leading to delayed closure of the wound, thereby contributing to the risk for DFU (31). Moreover, higher BMI triggers insulin resistance and inflammation, causing microvascular/neuropathic damage, higher plantar pressure, and impaired healing—thereby increasing DFU risk. (24).

It is interesting to note that the ML model showed similar predictive performance in both internal validation in U.S. NHANES data and external validation in a Chinese clinical cohort, with strong cross-population generalizability. This may be due to the fact that the model was based on several key predictive factors-e.r., urine albumin, peripheral neuropathy, abnormal blood glucose, and BMI — that have high generalizability and clinical interpretability across ethnicities, geographical locations, and healthcare system (32–34). These predictors are not only closely related to the fundamental pathophysiological processes of diabetic foot ulceration- e.g., nerve dysfunction, microvascular disease, immunosuppression, and impaired tissue repair—but are relatively easy to access and standardize, thereby avoiding the confounding effects of data quality and measurement variability (35). However, in light of model’s systematic miscalibration, we recommend localized model recalibration strategies aligned with local epidemiology, along with deployment strategies for it as an EHR-based clinical decision support system utilizing FHIR/CDS hooks for automated data entry. This will display ‘risk tier + key drivers + recommended actions’ output for clinicians, along with a ‘patient calculator, ‘ version control system, bias detection, and periodic recalibration system accordingly. Additionally, threshold-based risk stratification could guide clinical decisions: high-risk patients (threshold >20%) should receive frequent foot exams and specialist referrals, while moderate-risk patients (threshold 10%-20%) should be monitored with lifestyle interventions.

Nonetheless, this study has several limitations that should be noted. First, the NHANES data used to develop the models is cross-sectional in nature, limiting the extent to which causal or temporal relationships can be inferred. Second, the external validation may have introduced selection bias due to its study design and the significant recruitment time gap with the NHANES cohort, potentially affecting the comparability and generalizability of the results. Third, because the DFU outcome in NHANES was self-reported without adjudication, non-differential misclassification is possible and would be expected to primarily affect calibration. Fourth, sparse data in the low-probability range may introduce systematic miscalibration, reducing the reliability of low-risk decision thresholds. Finally, we acknowledge potential measurement bias in biochemical markers across cohorts, and the absence of imaging and plantar-pressure data further limits the completeness of DFU risk modeling.

## 5 Conclusion

In this study, machine-learning models performed strongly for DFU risk prediction, with the RF model showing the best overall performance across cohorts. Future prospective validation should adopt a pragmatic, real-time EHR pilot with continuous monitoring (discrimination, calibration, net benefit, equity) and periodic recalibration.

## Data Availability

The original contributions presented in the study are included in the article/**Supplementary Material**. Further inquiries can be directed to the corresponding authors.
