# Supplementary material for: Development and external validation of machine-learning based models to predict diabetic foot ulcer in diabetes population
Source: Front Endocrinol (Lausanne). 2025 Dec 15;16:1692917. doi: 10.3389/fendo.2025.1692917 (PMC12745231; doi:10.3389/fendo.2025.1692917)
Supplement: Supplementary file 1 [file DataSheet1.docx]

**SUPPLEMENTARY TABLES AND FIGURES**

**List of Supplementary Materials:**

Figure S1. Top 10 features ranked by permutation importance for the Random Forest (RF) model on the NHANES data set.

Figure S2. Confusion matrices of different cohorts. (a) Internal Validation Cohort; (b) External Validation Cohort.

Table S1. Variable mapping and description for NHANES data.

Table S2. Summary of variables with missing data in the NHANES dataset

Table S3 Final hyperparameters adopted in the six machine learning models

Table S4. Summary of Previous DFU Prediction Model

Figure S1. Top 10 features ranked by permutation importance for the Random Forest (RF) model on the NHANES dataset. Bars represent average permutation importance (higher bars = greater impact on model accuracy).

Clinical significance: Identifies the strongest contributors to risk in NHANES, informing variable selection, screening focus, and hypothesis generation.


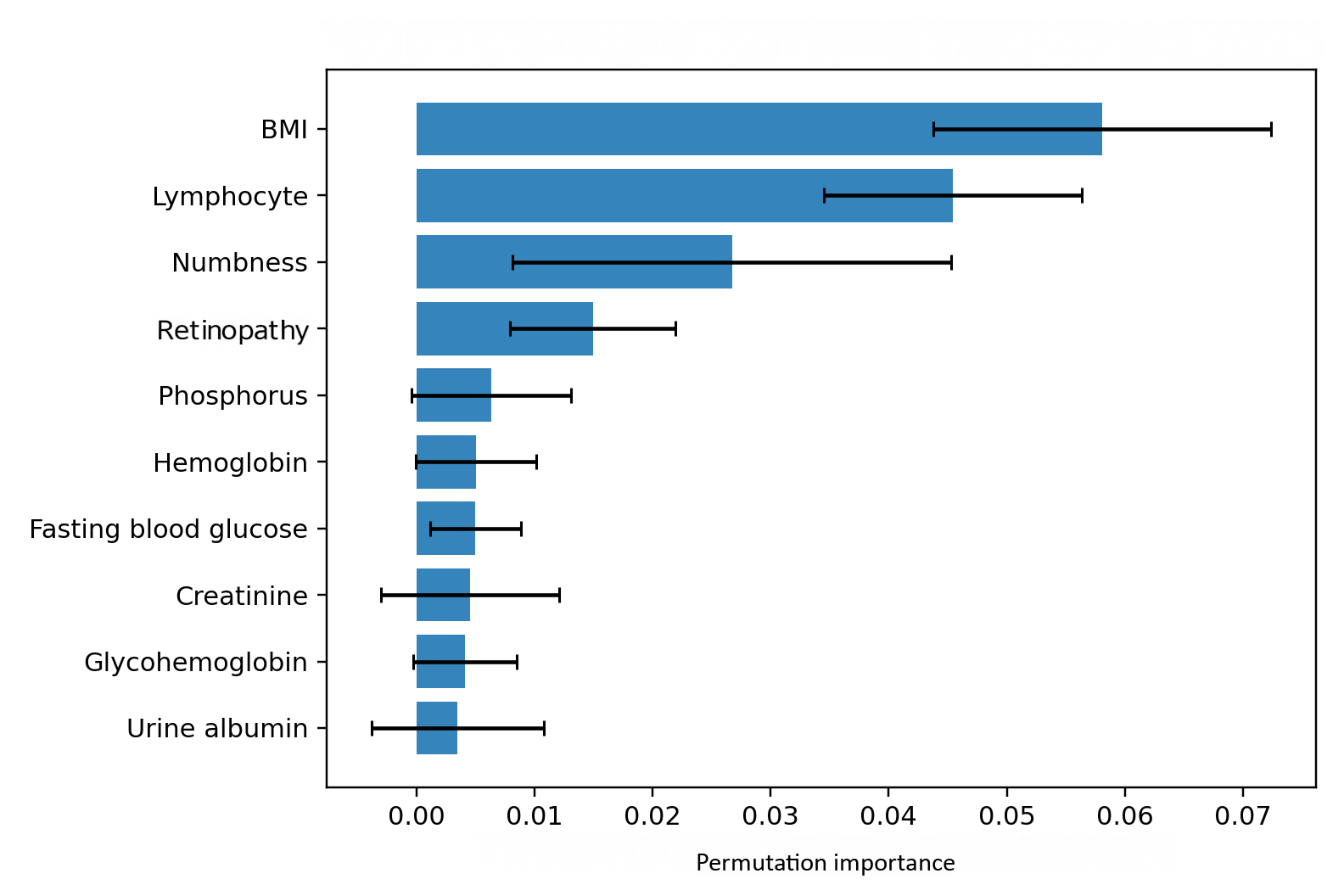


Figure S2. Confusion matrices of different cohorts. (a) Internal validation cohort; (b) external validation cohort. Cells show the numbers of true positives, false negatives, false positives, and true negatives; darker tiles indicate more cases.

Clinical significance: Highlights where the model makes errors (missed vs over-called cases), helping choose thresholds and prioritize follow-up or triage strategies.


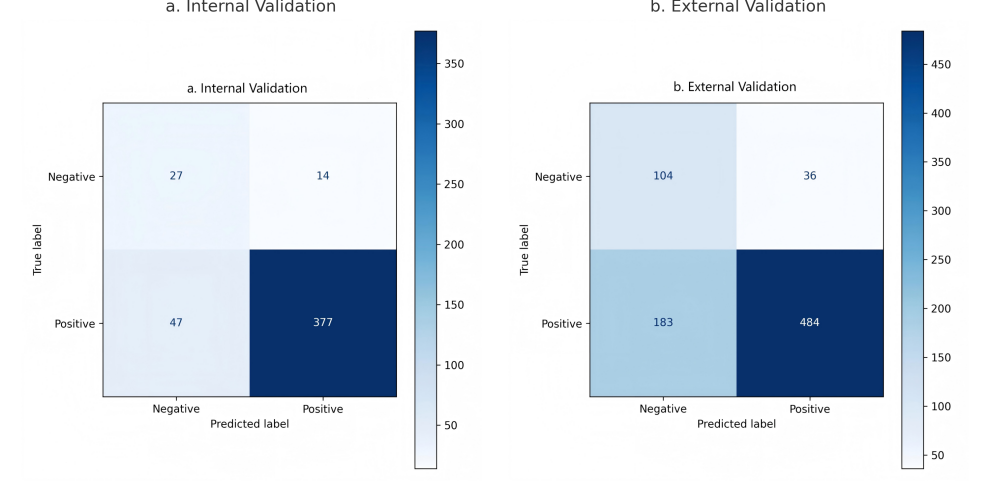


Table S1. Variable mapping and description for NHANES data.

| **NHANES Variable Code** | **Predictors** | **Description** |
| --- | --- | --- |
| SEQN | Respondent Sequence Number | Respondent sequence number |
| RIAGENDR | Gender | Gender of the sample person |
| RIDAGEYR | Age | Age at Screening Adjudicated - Recode |
| DIQ090 | Ulcer | Ulcer/sore not healed within 4 weeks |
| LBXGH | Glycohemoglobin | Glycohemoglobin (%) |
| LBDGLUSI | Fasting blood glucose | Plasma glucose: SI(mmol/L) |
| LBDSGLSI | Random blood glucose | Glucose, serum (mmol/L) |
| DIQ080 | Retinopathy | Diabetes affected eyes/had retinopathy |
| DIQ100 | Numbness in extremities | Numbness in hands/feet-past 3 mos |
| DIQ140 | Leg pain while walking | Pain in either leg while walking |
| MCQ160F | Stroke | Ever told you had a stroke |
| MCQ160C | Coronary heart disease | Ever told you had coronary heart disease |
| BPQ020 | Hypertension | Ever told you had high blood pressure |
| BPQ080 | Hypercholesterolemia | Doctor told you - high cholesterol level |
| KIQ022 | Renal dysfunction | Ever told you had weak/failing kidneys |
| ALQ101 | Alcohol | Had at least 12 alcohol drinks/1 yr? |
| SMQ020 | Smoking | Smoked at least 100 cigarettes in life |
| BMXBMI | Alcohol consumption | Body Mass Index (kg/m**2) |
| LBDTCSI | Total Cholesterol | Total Cholesterol( mmol/L) |
| LBDHDDSI | Direct HDL-Cholesterol | Direct HDL-Cholesterol (mmol/L) |
| URXUCR | Urine creatinine | Creatinine, urine (mg/dL) |
| URXUMA | Urine albumin | Albumin, urine (ug/mL) |
| LBXWBCSI | White blood cell count | White blood cell count (1000 cells/uL) |
| LBDNENO | Segmented neutrophils | Segmented neutrophils number |
| LBDLYMNO | Lymphocyte | Lymphocyte number |
| LBXHGB | Hemoglobin | Hemoglobin (g/dL) |
| LBXSKSI | Potassium | Potassium (mmol/L) |
| LBDSCASI | Total calcium | Total calcium (mmol/L) |
| LBDSPHSI | Phosphorus | Phosphorus (mmol/L) |
| LBXSAL | Albumin | Albumin (g/dL) |
| LBDSCRSI | Creatinine | Creatinine (umol/L) |
| LBXCRP | C-reactive protein | C-reactive protein(mg/dL) |

Table S2. Summary of variables with missing data in the NHANES dataset.

| **Variable** | **n missing** | **Missing (%)** | **Method** | **Imputed (n)^†^** |
| --- | --- | --- | --- | --- |
| Retinopathy | 410 | 21.08% | mode | 410 |
| Hypertension | 2 | 0.11% | mode | 2 |
| Hypercholesterolemia | 259 | 13.95% | mode | 259 |
| Alcohol consumption | 231 | 12.44% | mode | 231 |
| Glycohemoglobin | 191 | 10.29% | median | 191 |
| Fasting blood glucose | 1038 | 55.90% | median | 1038 |
| Random blood glucose | 747 | 40.23% | median | 747 |
| body mass index | 217 | 11.69% | median | 217 |
| Total Cholesterol | 231 | 12.44% | median | 231 |
| Direct HDL Cholesterol | 232 | 12.49% | median | 232 |
| Urine creatinine | 196 | 10.55% | median | 196 |
| Urine albumin | 196 | 10.55% | median | 196 |
| white blood cell count | 188 | 10.12% | median | 188 |
| Segmented neutrophils | 205 | 11.04% | median | 205 |
| Lymphocyte | 205 | 11.04% | median | 205 |
| Hemoglobin | 188 | 10.12% | median | 188 |
| Potassium | 237 | 12.76% | median | 237 |
| Total calcium | 237 | 12.76% | median | 237 |
| Phosphorus | 237 | 12.76% | median | 237 |
| Albumin | 237 | 12.76% | median | 237 |
| Creatinine | 237 | 12.76% | median | 237 |
| C-reactive protein | 222 | 11.95% | median | 222 |

Table S3 Final hyperparameters adopted in the five machine learning models

| Model | CV folds | Stratified | Seed | Search method | Parameter space (full) | Hyperparameter |
| --- | --- | --- | --- | --- | --- | --- |
| Random Forest | 5 | Yes | 42 | Grid | n_estimators {200, 500, 800, 1000}; max_depth {None, 8, 12, 16}; min_samples_split {2, 5, 10}; min_samples_leaf {1, 2, 4}; max_features {sqrt, log2}; bootstrap {True, False}; class_weight balanced | n_estimators=800, max_depth=12, min_samples_split=5, min_samples_leaf=2, max_features="sqrt", bootstrap=True, class_weight="balanced", random_state=42 |
| Logistic Regression | 5 | Yes | 42 | Grid | penalty {l2}; C ∈ [0.001, 10]; solver {liblinear, saga, lbfgs}; max_iter 10,000; class_weight balanced | penalty="l2", solver="lbfgs", C=0.5, max_iter=10,000, fit_intercept=True, class_weight="balanced", random_state=42 |
| K-Nearest Neighbors | 5 | Yes | 42 | Grid | n_neighbors {3, 5, 7, 9, 11, 15}; weights {uniform, distance}; metric minkowski; p {1, 2}; leaf_size {20, 30, 40} | n_neighbors=11, weights="distance", p=2 (Euclidean), metric="minkowski", leaf_size=30 |
| Support Vector Machine | 5 | Yes | 42 | Grid | kernel {linear, rbf}; C {0.1, 1, 10, 100}; gamma {scale, 0.01, 0.03, 0.1} (RBF); class_weight balanced; probability True | kernel="rbf", C=10, gamma=0.03, probability=True, class_weight="balanced", random_state=42 |
| XGBoost | 5 | Yes | 42 | Randomized (100) → narrowed grid | n_estimators [300–1000]; learning_rate {0.01, 0.05, 0.1}; max_depth {3, 4, 5, 7}; subsample {0.7, 0.9, 1.0}; colsample_bytree {0.6, 0.8, 0.9, 1.0}; min_child_weight {1, 3, 5}; gamma {0, 0.1, 0.3}; reg_lambda {1, 5, 10}; reg_alpha {0, 0.5, 1.0}; scale_pos_weight tuned | n_estimators=400, learning_rate=0.05, max_depth=4, subsample=0.9, colsample_bytree=0.9, min_child_weight=3, reg_lambda=1.0, reg_alpha=0.0, gamma=0.0, tree_method="hist", objective="binary:logistic", scale_pos_weight = (neg/pos from train split), random_state=42 |

Table S4. Summary of Previous DFU Prediction Models

| Study | Population | Predictor | Model | AUC |
| --- | --- | --- | --- | --- |
| Lv, 2023 | type 2 diabetes, older | BMI, bnormal foot skin color, foot arterial pulse, callus, history of ulcer | conventional | 0.79 |
| Chen, 2021 | diabetes | sex, BMI, HbA1c, smoker, DN, DR, DPN, intermittent claudication, foot care | conventional | 0.85 |
| Shao, 2023 | diabetes, older | age, presence of peripheral neuropathy, history of smoking, duration of disease, serum lactate dehydrogenase, high density cholesterol | conventional | 0.84 |
| Wang, 2024 | diabetes | cardiovascular disorders, peripheral artery disease, and neurological damage,  lower limb varicose veins, history of cerebral infarction, blood urea nitrogen, GFR | AutoML | 0.76 |
| Wang, 2025 | Diabetes with ASO | BMI, hypertension, coronary heart disease, diabetes nephropathy, calf artery occlusion times, insulin injection to control blood sugar, age, daily smoking times, diastolic blood pressure, CRP | ML ensemble | 0.97 |
| BMI, body mass index; DN, diabetic nephropathy; DR, diabetic retinopathy; DPN, diabetic peripheral neuropathy; GFR, glomerular filtration rate; ASO, arteriosclerotic occlusion; AutoML, Combining multiple gaussian weighted classifiers, random forests, and support vector machine; CRP, C-reactive protein; ML ensemble, LASSO, random forests, and support vector machine. | | | | |
